# Comprehensive analysis of peroxiredoxins expression profiles and prognostic values in breast cancer

**DOI:** 10.1186/s40364-019-0168-9

**Published:** 2019-08-06

**Authors:** Jie Mei, Leiyu Hao, Xiaorui Liu, Guangshun Sun, Rui Xu, Huiyu Wang, Chaoying Liu

**Affiliations:** 1 Department of Oncology, Wuxi People’s Hospital Affiliated to Nanjing Medical University, Wuxi, 214023 China; 2 Department of Physiology, Nanjing Medical University, Nanjing, 211166 China; 3 School of Pediatrics, Nanjing Medical University, Nanjing, 211166 China; 4 Department of General Surgery, Wuxi People’s Hospital Affiliated to Nanjing Medical University, Wuxi, 214023 China

**Keywords:** PRDX, Bioinformatic analysis, Gene expression, Prognostic, Breast cancer

## Abstract

**Background:**

The peroxiredoxins (PRDXs) gene family has been demonstrated to participate in carcinogenesis and development of numerous cancers and the prognostic values in several cancers have been evaluated already. Purpose of our research is to explore the expression profiles and prognostic values of PRDXs in breast cancer (BrCa).

**Methods:**

The transcriptional levels of PDRX family members in primary BrCa tissues and their association with intrinsic subclasses were analyzed using UALCAN database. Then, the genetic alterations of PDRXs were examined by cBioPortal database. Moreover, the prognostic values of PRDXs in BrCa patients were investigated via the Kaplan-Meier plotter.

**Results:**

The transcriptional levels of most PRDXs family members in BrCa tissues were significantly elevated compared with normal breast tissues. Meanwhile, dysregulated PRDXs expression was associated with intrinsic subclasses of BrCa. Besides, copy number alterations (CNA) of PRDXs positively regulated their mRNA expressions. Furthermore, high mRNA expression of PRDX4/6 was significantly associated with poor overall survival (OS) in BrCa patients, while high mRNA expression of PRDX3 was notably related to favorable OS. Simultaneously, high mRNA expression of PRDX1/2/4/5/6 was significantly associated with shorter relapse-free survival (RFS) in BrCa patients, while high mRNA expression of PRDX3 was notably related to favorable RFS. In addition, the prognostic value of PRDXs in the different clinicopathological features based on intrinsic subclasses and chemotherapeutic treatment of BrCa patients was further assessed in the KM plotter database.

**Conclusion:**

Our findings systematically elucidate the expression profiles and distinct prognostic values of PRDXs in BrCa, which might provide novel therapeutic targets and potential prognostic biomarkers for BrCa patients.

## Background

Breast cancer (BrCa) has the highest morbidity among all female’s cancers worldwide, which may cause 41,760 cancer-related deaths in the United States (US) in 2019 according to the prediction by the American Cancer Society (ACS) [1]. Being a multifaceted disease, BrCa can be classified into various subclasses based on the expression status of estrogen receptor (ER), progesterone receptor (PR), epidermal growth factor receptor 2 (HER2) and antigen ki-67 (Ki-67), which suggest different therapeutic guidance and prognostic implications for BrCa patients [2]. Although the constant amelioration of comprehensive therapies for BrCa has significantly decreased the mortality of BrCa in recent years, it is still necessary to further explore the potential mechanism of oncogenesis and progression of BrCa.

Peroxiredoxins (PRDXs), a family of antioxidant enzymes in eukaryotes, containing six isoforms (PRDX1, PRDX2, PRDX3, PRDX4, PRDX5, and PRDX6), which catalyze the reduction reaction of peroxide and maintain the balance of intracellular hydrogen peroxide (H_2_O_2_) levels [3]. As momentous regulators in diverse signaling pathways, PRDXs are of great significance to the signal transduction and cells metabolism [4]. Expression of PRDXs will be significantly upregulated when cells are under oxidative stress conditions. Several researches have suggested that overexpression of PRDXs may play dichotomous role in oncogenesis of tumors, where they could either stimulate the progression of cancers or suppress the development of cancers [5]. An increasing number of studies have observed the preliminary functions and ambiguous prognostic values of PRDXs in cancerous diseases [6–9]. However, the expression profiles and prognostic values of PRDXs in BrCa samples are still elusive up to now. Our research aims to explore the differential expression and potential roles of PRDXs in BrCa.

The Cancer Genome Atlas (TCGA) program, which launched by the US National Cancer Institute (NCI) and the National Human Genome Research Institute (NHGRI), attempts to sequence the entire genome of more than 10,000 tumor samples and to distinguish the genetic changes specific for each cancer [10–12]. Along with the successful implementation of the TCGA project, massive genomic information is accumulating exponentially. Over the past few years, many interactive and user-friendly online platforms based on the TCGA database greatly elevate the efficiency of TCGA database analysis and increasing amounts of tumor biomarkers have been identified on the strength of these websites [13, 14].

Therefore, in the current research, we first compared the transcriptional levels of PRDXs in BrCa and adjacent breast tissues using UALCAN database. In addition, the cBioPortal database was used to analyze the genetic alterations of PDRXs and the correlation with transcriptional levels. Moreover, the Kaplan-Meier plotter database was used to assess the prognostic effects of PRDXs mRNA expression in patients with BrCa. Overall, our research preliminarily but systematically characterizes the expression profiles of PRDXs in BrCa and reveals that the detection of the PRDXs expression status of BrCa patients may be valuable and potential biomarkers for prognostic assessment.

## Materials and methods

### Gene expression analysis via UALCAN

UALCAN (http://ualcan.path.uab.edu/) is an online open-access platform based on level 3 RNA-seq and clinical information from TCGA database [15]. It can be used to analyze relative transcriptional levels of potential genes of interest between cancerous and paired normal tissues and association of the transcriptional levels with clinicopathologic features. In the current study, UALCAN was applied to analyze the transcriptional levels of PDRXs family members in primary BrCa tissues and their association with intrinsic subclasses. All the BrCa cases available on UALCAN were included in our research.

### Data-mining analysis based on cBioPortal

cBioPortal (www.cbioportal.org/) is a user-friendly, interactive website resource and provides visualization, analysis, and download of large-scale cancer genomics datasets [16, 17]. In the current study, we analyzed the genetic alterations of PDRXs family members, which contained mutations and putative copy-number alterations (CNA) from GISTIC. Furthermore, we download the data of putative copy-number alterations and mRNA expression z-Scores to evaluate the association between various CNAs and transcriptional levels of PRDXs. Tumor samples with RNA-seq and CAN data on cBioPortal were included in our research which contains total 1076 BrCa samples.

### Survival analysis by Kaplan-Meier plotter

Kaplan-Meier Plotter (KM Plotter, http://kmplot.com/analysis/) is an online database containing gene expression profiles and survival information of cancer patients [18]. The prognostic values of PRDXs (PRDX1, PRDX2, PRDX3, PRDX4, PRDX5, and PRDX6) at mRNA level in BrCa was analyzed using all BrCa samples available on KM Plotter. The patients’ cohorts were split at the median expression of each PRDXs mRNA level. The subgroup analysis of the prognostic value of PRDXs in BrCa patients was further performed according to intrinsic subclasses and different regimens of chemotherapy. All cohorts were compared with Kaplan-Meier survival plots. Hazard ratio (HR), 95% confidence interval (95% CI), and log-rank *P* value were calculated and displayed online.

### Statistical analysis

All statistical analyses were performed on the bioinformatics database online or using SPSS 25.0 software (Chicago, IL). The differential mRNA expression of PRDXs in BrCa tissues was analyzed by Student’s *t*-test. Kaplan-Meier survival plots were generated with survival curves compared by log-rank test. For all analyses, Differences were considered statistically significant if *P* values were less than 0.05.

## Results

### Transcriptional levels of PRDXs in BrCa samples

**Fig. 1:**
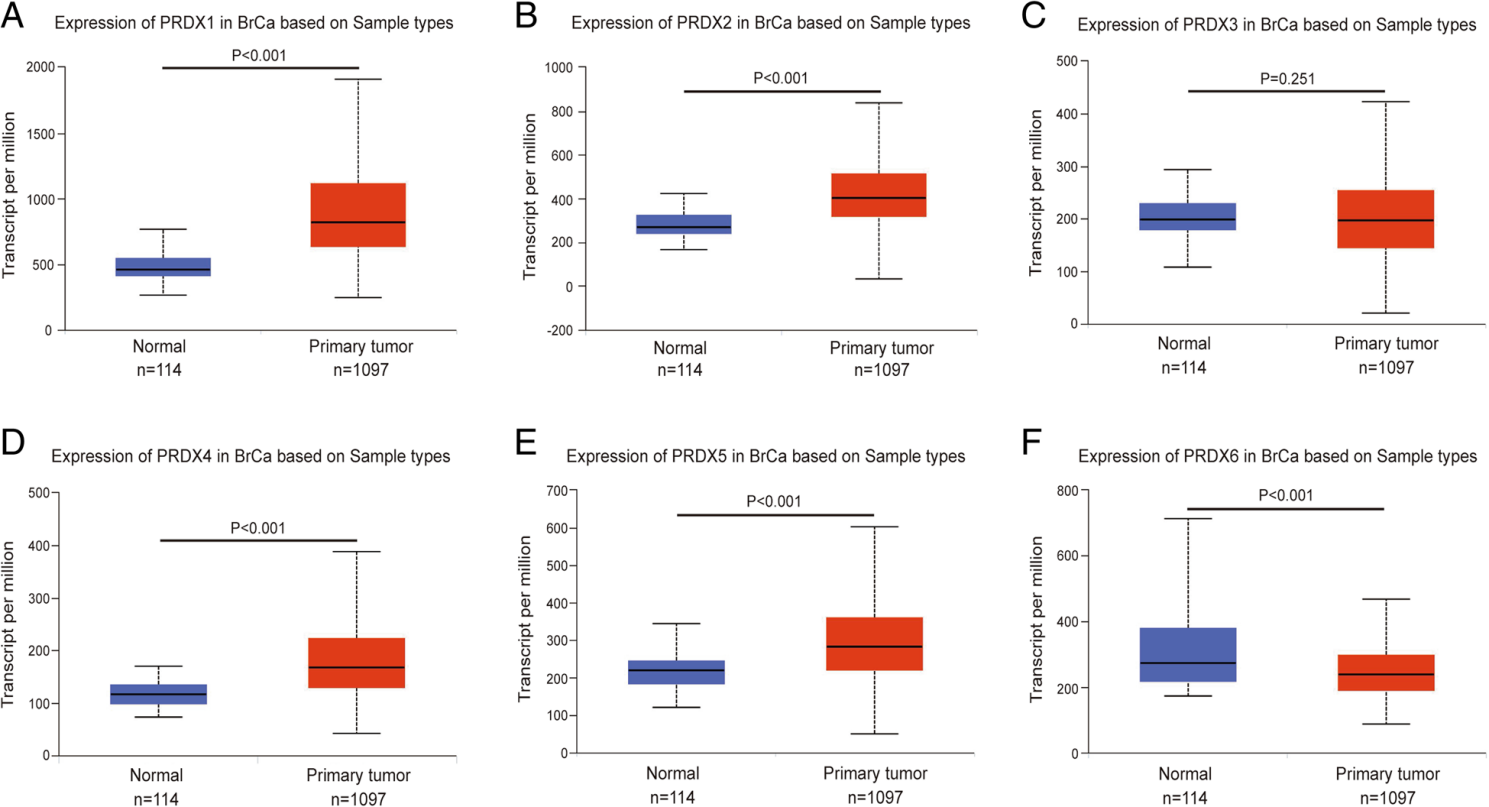
Transcriptional levels of PRDXs in paracancerous and BrCa tissues. Comparison of PRDX1, PRDX2, PRDX3, PRDX4, PRDX5, PRDX6 mRNA expression in paracancerous (*n* = 114) and BrCa (*n* = 1097) tissues in TVGA dataset based on data mining via UALCAN. **a**, **b**, **d**, **e**) The transcriptional level of PRDX1, PRDX2, PRDX4, and PRDX5 was significantly upregulated in BrCa tissues compared with paracancerous tissues. **c** The transcriptional level of PRDX3 showed a non-significant difference in BrCa tissues compared with paracancerous tissues. **f** The transcriptional level of PRDX6 was significantly downregulated in BrCa tissues compared with paracancerous tissues

In order to evaluate the exact expression profiles of PRDXs members in BrCa patients, the differential transcriptional levels of PRDX family members between BrCa and paired normal breast tissue was evaluated by UALCAN database. As shown in Fig. 1, the transcriptional level of PRDX1 (Fig. 1a, *P* < 0.001), PRDX2 (Fig. 1b, *P* < 0.001), PRDX4 (Fig. 1d, *P* < 0.001), and PRDX5 (Fig. 1e, *P* < 0.001) was significantly upregulated in BrCa tissues compared with paracancerous tissues. However, the transcriptional level of PRDX3 (Fig. 1c, *P* = 0.251) showed a non-significant difference in BrCa tissues compared with paracancerous tissues. Besides, the transcriptional level of PRDX6 was significantly downregulated in BrCa tissues compared with paracancerous tissues (Fig. 1f, *P* < 0.001).

### Transcriptional levels of PRDXs in different BrCa subclasses

**Fig. 2:**
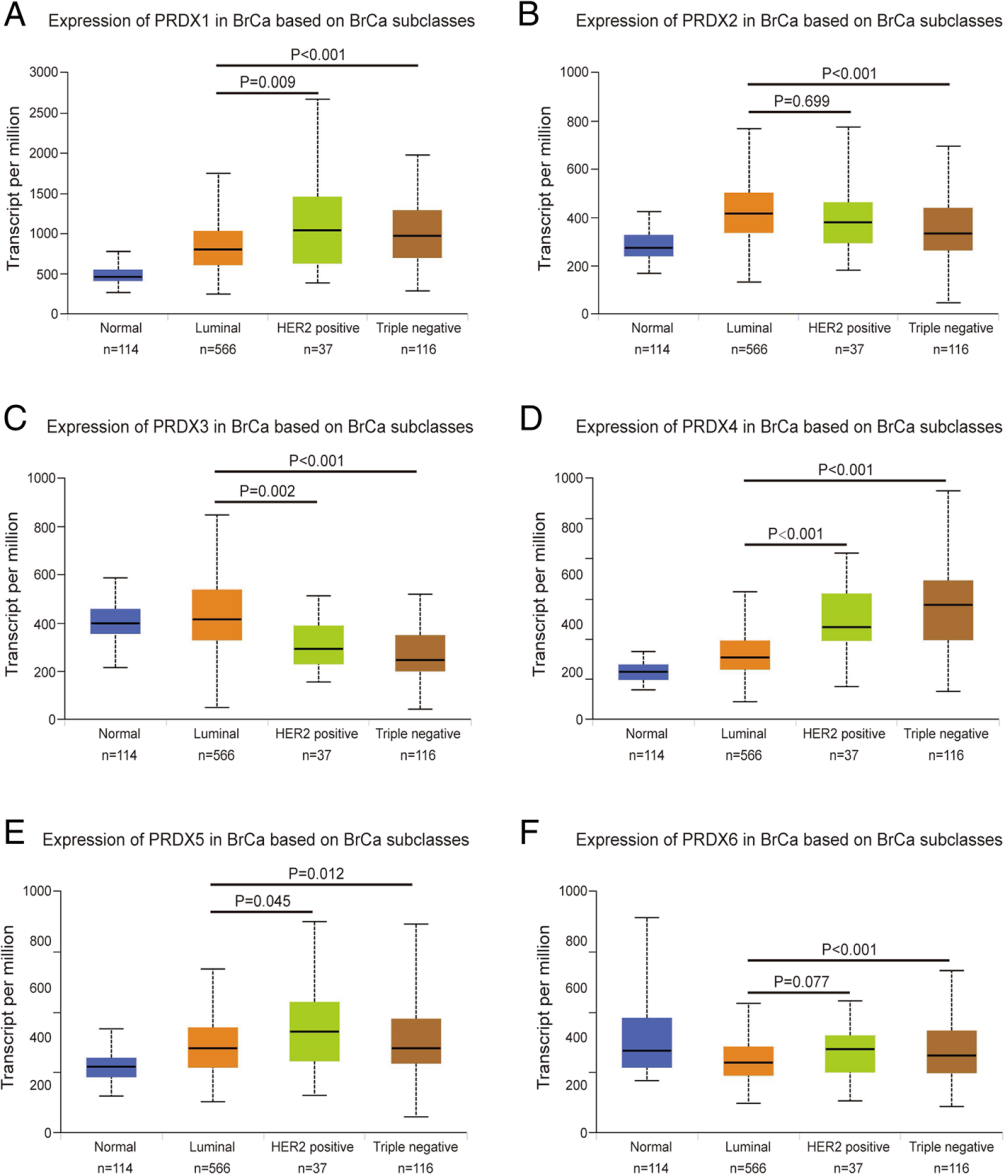
Transcriptional levels of PRDXs in various BrCa subclasses. The transcriptional level of PRDXs in BrCa patients with different subclasses, PRDXs mRNA was significantly downregulated (PRDX3) or upregulated (other PRDXs) in HER2-positive and triple-negative BrCa tissues compared with luminal BrCa tissues. **a** PRDX1. **b** PRDX2. **c** PRDX3. **d** PRDX4. **e** PRDX5. **f** PRDX6

Classification of intrinsic subclasses is helpful in the prediction of therapeutic response and prognosis of BrCa [19]. So, we next compared the differential transcriptional levels of PRDX family members according to different intrinsic subclasses of BrCa. As shown in Fig. 2, mRNA expressions of PRDXs family members were significantly correlated with intrinsic subclasses of BrCa. Patients who were with HER2-positive and triple-negative subclasses BrCa tended to express higher PRDXs (exclude PRDX2, PRDX3) mRNA, while express lower PRDX2 and PRDX3 mRNA. The highest mRNA expressions of PRDX1/5/6 were found in HER2-positive tissues (Fig. 2a, e, f), and the highest mRNA expressions of PRDX4 were found in triple-negative tissues (Fig. 2d). Besides, the lowest mRNA expressions of PRDX2/3 were found in triple-negative tissues (Fig. 2b, c). Taken together, these findings above revealed that transcriptional levels of PRDXs family members were significantly correlated with intrinsic subclasses in BrCa patients.

### Genetic alterations of PRDXs in BrCa samples

**Fig. 3:**
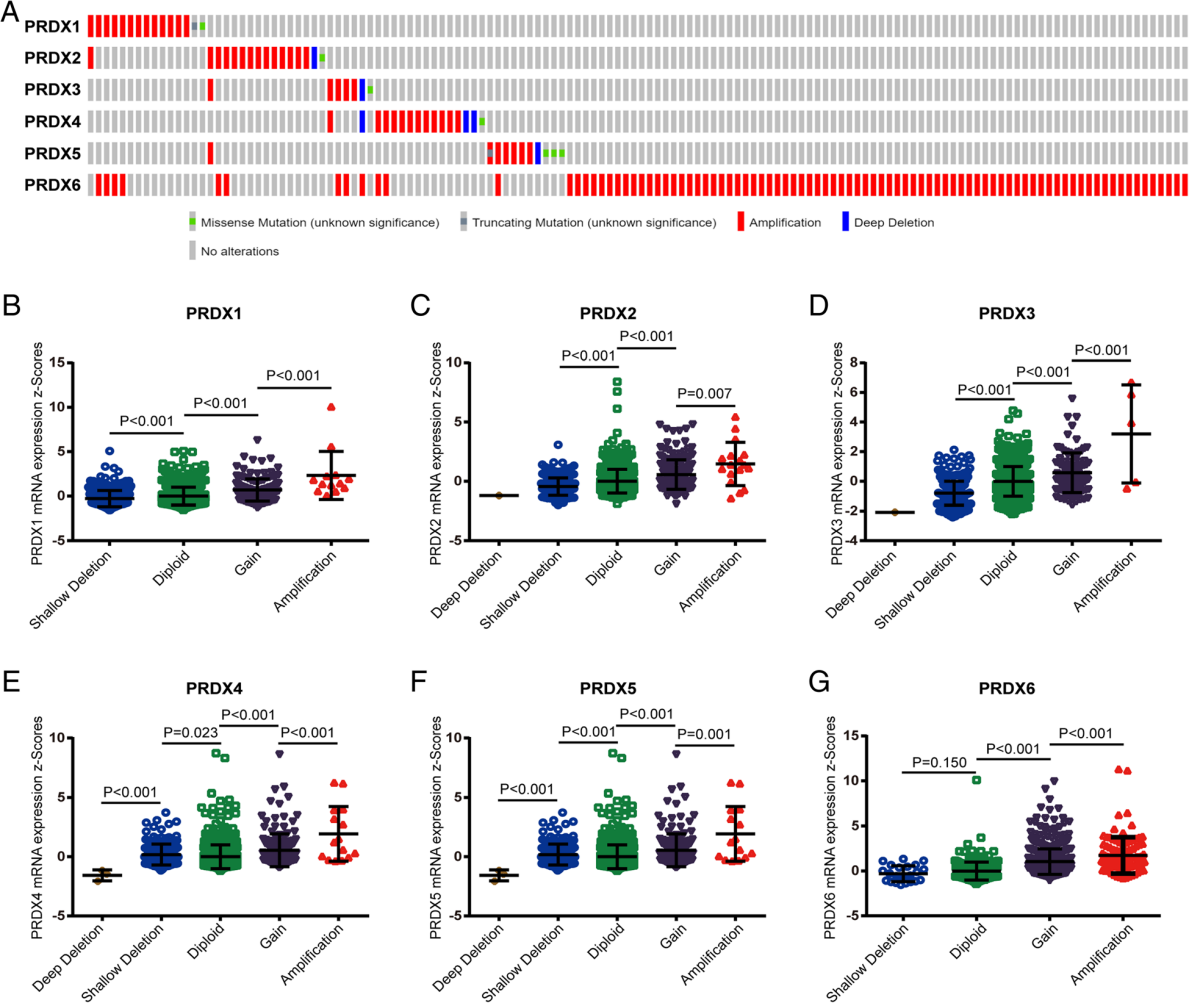
Correlation between the genetic alterations of PRDXs and mRNA levels in BrCa tissues. **a** Oncoprint in cBioPortal database exhibited the proportion and distribution of specimens with genetic alterations in PRDXs. The Figure was cropped on the right to exclude specimens without any alterations. **b**-**g** Copy gain (gain and amplification) of PRDXs was associated with notably upregulated PRDXs mRNA levels compared with the copy-neutral (diploid) and copy-loss (shallow deletion and deep deletion) cases. **b** PRDX1. **c** PRDX2. **d** PRDX3. **e** PRDX4. **f** PRDX5. **g** PRDX6

DNA copy number alterations (CNA) are most common genetic alterations which participates in oncogenesis of cancers via regulating cancer-related gene expression [20–22]. In the fact that most PRDX family members was dysregulated in BrCa tissues, we speculated that DNA CNA may regulate the transcriptional levels of PRDXs. Next, we analyzed genetic alteration in PRDXs and correlations with their mRNA expressions based on cBioPortal website. As shown in Fig. 3a and Table 1, low amplification rate of PRDXs was found in BrCa patients. However, although copy gain (gain and amplification) of PRDXs was not frequent, it was still associated with notably upregulated PRDXs mRNA levels compared with the copy-neutral (diploid) and copy-loss (shallow deletion and deep deletion) cases (Fig. 3b-g). To conclude, the results suggested that PRDX mRNA expressions were regulated by their DNA copy number alterations.

**Table 1:** Frequency and proportion of genetic alterations of PRDXs in BrCa

PRDXs	Deep deletion	Shallow deletion	Dipliod	Gain	Amplification
PRDX1	0 (0.00%)	297 (27.60%)	623 (57.90%)	143 (13.29%)	13 (1.21%)
PRDX2	1 (0.09%)	226 (21.00%)	646 (60.04%)	186 (17.29%)	17 (1.58%)
PRDX3	1 (0.09%)	321 (29.83%)	651 (60.50%)	98 (9.11%)	5 (0.46%)
PRDX4	3 (0.28%)	184 (17.10%)	706 (65.61%)	168 (15.61%)	15 (1.39%)
PRDX5	1 (0.09%)	242 (22.49)	657 (61.06%)	167 (15.52%)	9 (0.84%)
PRDX6	0 (0.00%)	24 (2.23%)	262 (24.35%)	689 (64.03%)	101 (9.39%)

### Prognostic values of PRDXs mRNA expression in all BrCa samples

**Fig. 4:**
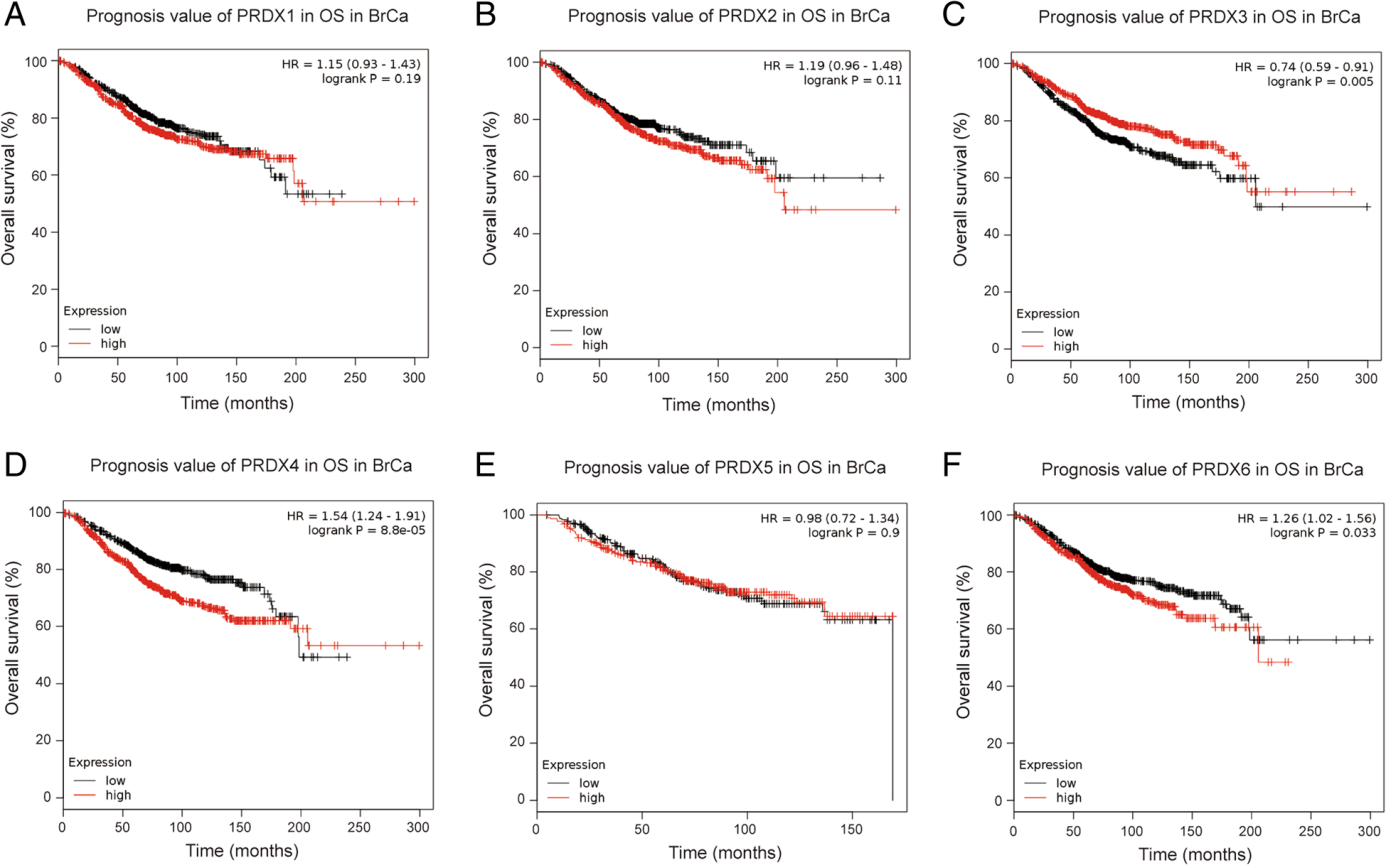
Prognostic value of PRDXs mRNA in all BrCa patients (OS). OS curves were plotted to evaluate the prognostic value of PRDXs mRNA expression. **a** PRDX1 (208680_at). **b** PRDX2 (39729_at). **c** PRDX3 (201619_at). **d** PRDX4 (201923_at). **e** PRDX5 (1560587_s_at). **f** PRDX6 (200845_s_at)

**Fig. 5:**
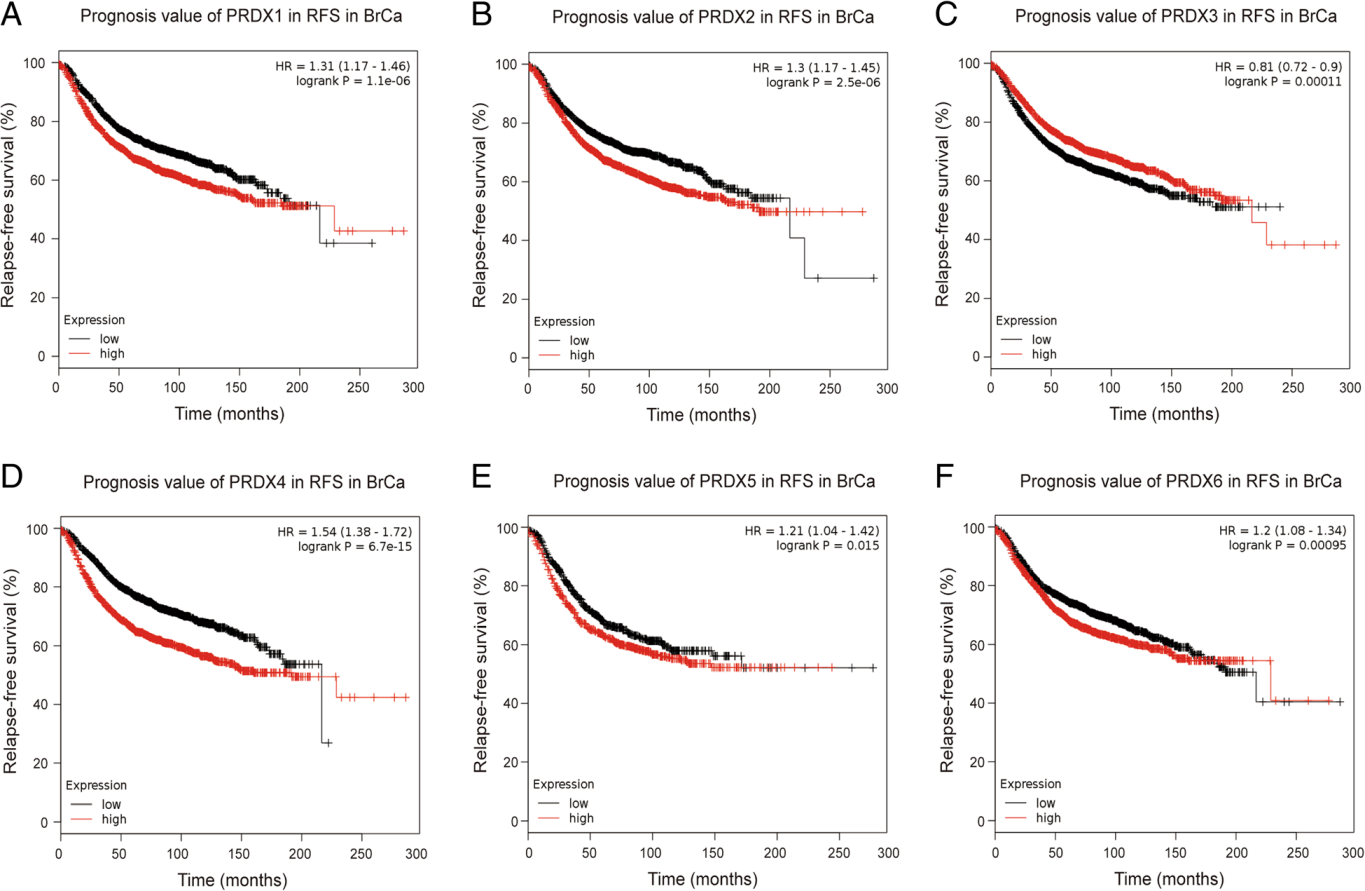
Prognostic value of PRDXs mRNA in BrCa patients (RFS). RFS curves were plotted to evaluate the prognostic value of PRDXs mRNA expression. **a** PRDX1 (208680_at). **b** PRDX2 (39729_at). **c** PRDX3 (201619_at). **d** PRDX4 (201923_at). **e** PRDX5 (1560587_s_at). **f** PRDX6 (200845_s_at)

Further, we employed KM plotter to evaluate the prognostic values of PRDX family members. As shown in Fig. 4 and Fig. 5, high mRNA expression of PRDX4 (HR = 1.54, 95% CI: 1.24–1.91, *P* < 0.001), and PRDX6 (HR = 1.26, 95% CI: 1.02–1.56, *P* = 0.033) were significantly associated with poor overall survival (OS) of BrCa patients, while high mRNA expression of PRDX3 was notably related to favorable OS of BrCa patients (HR = 0.74, 95% CI: 0.59–0.91, *P* = 0.005). However, other PRDXs mRNA expression showed a null association with prognosis of BrCa patients.

We next analyzed the associations between PRDXs mRNA expression and RFS of BrCa patients and found that high mRNA expression of PRDX1 (HR = 1.31, 95% CI: 1.17–1.46, *P* < 0.001), PRDX2 (HR = 1.30, 95% CI: 1.17–1.45, *P* < 0.001), PRDX4 (HR = 1.54, 95% CI: 1.38–1.72, *P* < 0.001), PRDX5 (HR = 1.21, 95% CI: 1.04–1.42, *P* = 0.015) and PRDX6 (HR = 1.20, 95% CI: 1.08–1.34, *P* < 0.001) were significantly associated with shorter relapse-free survival (RFS) of BrCa patients, while high mRNA expression of PRDX3 was notably related to favorable RFS of BrCa patients (HR = 0.81, 95% CI: 0.72–0.90, *P* < 0.001). Overall, the findings above implied that mRNA expressions of PRDX3/4/6 were remarkably correlated with BrCa patients’ both OS and RFS, which might be identified as promising biomarkers to predict the survival of BrCa patients.

### Prognostic values of PRDXs mRNA in different BrCa subclasses

To further analyze the association of PRDXs mRNA expression with various BrCa subclasses, we detected the survival effects of PRDX family members in 4 subclasses of BrCa patients, including basal-like, luminal A, luminal B, and HER2 positive BrCa. As shown in Table 2, for PRDX1 (HR = 1.52, 95% CI: 1.06–2.17, *P* = 0.022), PRDX4 (HR = 1.58, 95% CI: 1.10–2.27, *P* = 0.012), and PRDX6 (HR = 1.49, 95% CI: 1.04–2.13, *P* = 0.027), high mRNA expression was associated with unfavorable OS in luminal A BrCa patients, respectively. Besides, high mRNA expression of PRDX4 (HR = 1.55, 95% CI: 1.06–2.27, *P* = 0.023) predicted poor OS in luminal B BrCa patients as well.

**Table 2:** Association between prognostic value of PRDXs mRNA expression and different subclasses in BrCa

Subclasses	Cases	HR	95% CI	*P* value	Cases	HR	95% CI	*P* value
	OS				RFS			
PRDX1								
Basal-like	241	0.66	0.40–1.09	0.104	618	1.02	0.80–1.32	0.850
Luminal A	611	1.52	1.06–2.17	0.022	1933	1.23	1.06–1.49	0.009
Luminal B	433	0.98	0.68–1.43	0.956	1149	1.20	0.99–1.46	0.060
HER2 positive	117	1.27	0.66–2.42	0.470	251	1.31	0.89–1.92	0.170
PRDX2								
Basal-like	241	1.14	0.70–1.85	0.613	618	1.25	0.97–1.61	0.078
Luminal A	611	1.42	0.99–2.02	0.056	1933	1.23	1.04–1.46	0.016
Luminal B	433	0.99	0.67–1.44	0.960	1149	1.67	1.37–2.03	< 0.001
HER2 positive	117	0.93	0.49–1.77	0.821	251	1.24	0.85–1.83	0.264
PRDX3								
Basal-like	241	0.77	0.47–1.26	0.299	618	0.77	0.60–1.00	0.046
Luminal A	611	0.74	0.52–1.05	0.090	1933	0.87	0.73–1.03	0.112
Luminal B	433	1.02	0.70–1.48	0.919	1149	1.17	0.96–1.41	0.115
HER2 positive	117	0.59	0.30–1.15	0.118	251	0.73	0.49–1.07	0.102
PRDX4								
Basal-like	241	0.67	0.41–1.11	0.119	618	1.04	0.81–1.33	0.784
Luminal A	611	1.58	1.10–2.27	0.012	1933	1.56	1.31–1.85	< 0.001
Luminal B	433	1.55	1.06–2.27	0.023	1149	1.56	1.28–1.89	< 0.001
HER2 positive	117	0.41	0.20–0.83	0.010	251	0.83	0.57–1.23	0.358
PRDX5								
Basal-like	153	1.09	0.58–2.06	0.790	360	1.71	1.23–2.37	0.001
Luminal A	271	1.09	0.66–1.81	0.725	841	1.19	0.93–1.53	0.164
Luminal B	129	0.58	0.29–1.15	0.117	407	0.95	0.70–1.29	0.752
HER2 positive	73	1.59	0.71–3.54	0.253	156	1.34	0.85–2.11	0.213
PRDX6								
Basal-like	241	1.08	0.66–1.76	0.761	618	1.01	0.78–1.29	0.963
Luminal A	611	1.49	1.04–2.13	0.027	1933	1.29	1.08–1.53	0.004
Luminal B	433	0.95	0.65–1.37	0.771	1149	1.27	1.05–1.54	0.015
HER2 positive	117	0.87	0.46–1.66	0.675	251	0.93	0.63–1.36	0.708

Moreover, we also analyzed the associations between PRDXs mRNA expression and RFS of various subclasses BrCa patients and the results indicated that high expressions of PRDX1 (luminal A: HR = 1.23, 95% CI: 1.06–1.49, *P* = 0.009), PRDX2 (luminal A: HR = 1.23, 95% CI: 1.04–1.46, *P* = 0.016; luminal B: HR = 1.67, 95% CI: 1.37–2.03, *P* < 0.001), PRDX4 (luminal A: HR = 1.56, 95% CI: 1.31–1.85, *P* < 0.001; luminal B: HR = 1.56, 95% CI: 1.28–1.89, *P* < 0.001), PRDX6 (luminal A: HR = 1.29, 95% CI: 1.08–1.53, *P* = 0.004; luminal B: HR = 1.27, 95% CI: 1.05–1.54, *P* = 0.015) were correlated with worse RFS in luminal BrCa patients. In addition, low expression of PRDX3 (HR = 0.77, 95% CI: 0.60–1.00, *P* = 0.046) and high expression of PRDX5 (HR = 1.71, 95% CI: 1.23–2.37, *P* = 0.001) predicted unfavorable RFS in basal-like BrCa patients. Thus, these results suggested the roles of PRDXs as potential prognostic predictors in BrCa patients with different subclasses.

### Prognostic values of PRDXs mRNA in BrCa patients with diverse regimens of chemotherapy

Next, we also checked the prognostic effects of PRDX family members in BrCa patients with different chemotherapies, including adjuvant chemotherapy, neoadjuvant chemotherapy and non-chemotherapy. As shown in Table 3, high expression of PRDX1, PRDX2, PRDX3, and PRDX4 were significantly correlated with poor OS in BrCa patients with adjuvant chemotherapy. In addition, in BrCa patients who didn’t receive any chemotherapies, high expression PRDX1 (HR = 1.22, 95% CI: 1.03–1.44, *P* = 0.019), PRDX4 (HR = 1.37, 95% CI: 1.16–1.62, *P* < 0.001), PRDX6 (HR = 1.19, 95% CI: 1.00–1.40, *P* = 0.044) and low expression of PRDX3 (HR = 0.84, 95% CI: 0.71–1.00, *P* = 0.043) were associated with worse RFS. However, contrary to the prognostic effect of PRDX5 in total BrCa patients, high expression of PRDX5 (HR = 0.50, 95% CI: 0.30–0.81, *P* = 0.005) predicted better RFS in BrCa patients with adjuvant chemotherapy. Thus, these results suggested the roles of PRDXs as potential prognostic predictors in BrCa patients with different regimens of chemotherapy.

**Table 3:** Association between prognostic value of PRDXs mRNA expression and various chemotherapies in BrCa

Chemotherapies	Cases	HR	95% CI	*P* value	Cases	HR	95% CI	*P* value
	OS				RFS			
PRDX1								
Adjuvant chemotherapy	163	1.85	1.01–3.40	0.044	594	1.05	0.78–1.42	0.734
Neoadjuvant chemotherapy	156	0.74	0.34–1.60	0.448	223	1.19	0.69–2.03	0.539
Non-chemotherapy	549	1.13	0.79–1.60	0.502	1873	1.22	1.03–1.44	0.019
PRDX2								
Adjuvant chemotherapy	163	2.02	1.09–3.73	0.023	594	1.33	0.98–1.80	0.064
Neoadjuvant chemotherapy	156	1.41	0.66–3.02	0.375	223	1.24	0.72–2.16	0.438
Non-chemotherapy	549	1.14	0.80–1.62	0.483	1873	1.07	0.90–1.26	0.444
PRDX3								
Adjuvant chemotherapy	163	2.03	1.10–3.73	0.021	594	0.80	0.59–1.08	0.148
Neoadjuvant chemotherapy	156	1.05	0.49–2.23	0.906	223	0.95	0.55–1.65	0.851
Non-chemotherapy	549	0.79	0.56–1.12	0.185	1873	0.84	0.71–1.00	0.043
PRDX4								
Adjuvant chemotherapy	163	1.97	1.07–3.65	0.027	594	1.02	0.75–1.38	0.906
Neoadjuvant chemotherapy	156	0.68	0.31–1.48	0.325	223	1.19	0.69–2.07	0.529
Non-chemotherapy	549	1.28	0.90–1.82	0.177	1873	1.37	1.16–1.62	< 0.001
PRDX5								
Adjuvant chemotherapy	0	1.09	0.58–2.06	0.790	255	0.50	0.30–0.81	0.005
Neoadjuvant chemotherapy	107	0.67	0.24–1.89	0.446	111	1.22	0.58–2.57	0.595
Non-chemotherapy	0	0.58	0.29–1.15	0.117	243	0.66	0.38–1.14	0.131
PRDX6								
Adjuvant chemotherapy	163	0.86	0.48–1.56	0.625	594	1.10	0.81–1.49	0.532
Neoadjuvant chemotherapy	156	0.72	0.33–1.55	0.395	223	1.09	0.63–1.88	0.769
Non-chemotherapy	549	1.33	0.94–1.88	0.110	1873	1.19	1.00–1.40	0.044

## Discussion

Reactive oxygen species (ROS), including the superoxide radical, the hydroxyl radical, H_2_O_2_ and etc., are the most important type of free radicals which produces secondary toxic metabolic products, such as peroxynitrites and nitrogen oxides, posing a lethal threat to cells by damaging DNA [23, 24]. Peroxiredoxins, one of the most significant antioxidant enzyme systems that include SOD, CAT, and GPx, were significnatly upregulated under oxidative stress conditions and mainly participate in the defense against oxidative [25, 26]. Several studies have observed that imbalances between the generation of ROS and PRDXs in tumor cells could lead to oxidative stress and the induction of cell apoptosis [27].

It has been demonstrated that PRDXs expression was significantly dysregulated during carcinogenesis of cancers and played dichotomous roles in oncogenesis. Overexpression of PRDX1 in BrCa has been observed to be positively associated with tumor grade and acted as dominant role in management of exogeneous oxidative stress [28, 29]. PRDX2, has been reported to specifically regulate the oxidative and metabolic stress response of metastatic breast cancer cells in lungs. Besides, overexpressed PRDX2 participates in chemo-resistant in BrCa cells [30, 31]. The function of PRDX3 and PRDX4 in BrCa is largely ambiguous, Liu et al. reveals that downregulation of PRDX3 potentiates PP2-induced apoptosis in MCF-7 cells, which suggests the tumor suppressor role of PRDX3 [32]. PRDX4 has been demonstrated to mediate osteoclast activation by human BrCa cells and enhance the aggressive phenotype [33]. Last but not least, there is no available research about the exact function of PRDX5 and PRDX6 in BrCa, but overexpression of PRDX5 and prognostic values have been observed in numerous cancers, including ovarian cancer and endometrial cancer [34, 35]. As well, the tumor promoter role of PRDX6 in cancers has also been suggested in colorectal cancer, lung cancer and so on [36, 37]. Although several studies that investigate the role of PRDXs in BrCa have been published, little is known about individual PRDXs expression and their effects on survival of BrCa patients.

In the present study, we first investigated the differential transcriptional levels of PRDX family members between BrCa and adjacent tissues and the results showed that the transcriptional levels of PRDX1, PRDX2, PRDX4, and PRDX5 were significantly upregulated in BrCa tissues. Besides, the transcriptional level of PRDX6 was significantly downregulated in BrCa tissues. However, the transcriptional level of PRDX3 showed a non-significant difference in BrCa tissues compared with adjacent tissues. we also compared the differential transcriptional levels of PRDX family members according to different intrinsic subclasses of BrCa and found mRNA expressions of PRDXs family members were significantly correlated with intrinsic subclasses of BrCa.

DNA copy number alterations (CNA) are most common genetic alterations which affect carcinogenesis and development of cancers by regulating cancer-related gene expression [20–22]. When we used cBioPortal to inspect genetic alteration in PRDXs and correlations with their mRNA expressions, the results showed that copy gain (gain and amplification) of most PRDXs was not frequent in BrCa, but it was still associated with notable upregulated PRDXs mRNA levels. Amplification is a positive factor to upregulate gene expression [38, 39]. However, to our largely surprise, the copy-gain frequency of PRDX6 accounts for a large proportion in total BrCa samples, but a significant decrease expression of PRDX6 was exhibited in BrCa tissues. We speculated that amplification of PRDX6 gene may upregulate PRDX6 expression in BrCa tissues compared with paired breast tissues. However, limited amounts of normal tissues expression data were included in TCGA dataset, thus, the opposite phenomenon that PRDX6 amplification decreased transcriptional level was exhibited.

The Kaplan-Meier plotter is an online database which is available to assess the prognostic effect of genes expression on survival in designate cancers. The primary purpose of the tool is a meta-analysis-based biomarker assessment and a lot of prognostic biomarkers have been identified based on this platform [40–43]. It has been reported that overexpression PRDX6 participates in cisplatin resistance in ovarian cancer and predicts poor OS and PFS [8, 44]. Several researches also observed the promising prognostic values of PRDXs in lung cancer and endometrial cancer [7, 35, 45]. However, the prognostic values of PRDXs in BrCa patients are largely unknown. Here, we found that high mRNA expression of PRDX4/6 were significantly associated with poor OS of BrCa patients and high mRNA expression of PRDX1/2/4/5/6 were significantly associated with shorter RFS of BrCa patients, while high mRNA expression of PRDX3 was notably related to favorable OS and DFS, which suggests the tumor suppressor role of PRDX3 in BrCa. Besides, the prognostic values of PRDXs mRNA in different BrCa subclasses and in BrCa patients with diverse regimens of chemotherapy were also assessed and results suggested the potential roles of PRDXs in predicting prognosis of BrCa patients with various subclasses and different regimens of chemotherapy.

Although this study systematically demonstrates the prognostic value of PRDXs in breast cancer, this research has several limitations as well. The major limitation is that online database only provides the expression of PRDXs mRNA level, which may not fully represent the expression of PRDXs at the protein level. In further study, western blotting, immumohistochemical staining and other protein detection techniques will be applied to determinate the protein level of PRDXs in breast cancer. Furthermore, the possible mechanisms that PRDXs is involved in the tumorigenesis and progression of breast cancer need to be further studied. Besides, although the large sample analyses based on meta-analysis have some advantages, but some essential information form one single center may be missing, such as some therapeutic information.

## Conclusion

In summary, we systemically analyzed the expression profiles and prognostic values of PRDXs in BrCa. Our results revealed that PRDX1/2/4/5/6 might be the potential therapeutic targets for BrCa therapy, whereas PRDX3/4/6 were promising prognostic biomarkers for predicting OS and RFS of BrCa patients. Overall, our research provided a systematic insight into the heterogeneous and complex roles of PRDXs in the carcinogenesis of BrCa.

## Data Availability

All data are included in the article.

## Abbreviations

ACS: The American Cancer Society
BrCa: Breast cancer
CAN: Copy-number alterations
CI: Confidence interval
ER: Estrogen receptor
HER2: Epidermal growth factor receptor 2
HR: Hazard ratio
Ki-67: Antigen ki-67
NCI: US National Cancer Institute
NHGRI: The National Human Genome Research Institute
OS: Overall survival
PR: Progesterone receptor
PRDX: Peroxiredoxin
RFS: Relapse-free survival
TCGA: The Cancer Genome Atlas
