# Correction: Comprehensive analysis of peroxiredoxins expression profiles and prognostic values in breast cancer

**DOI:** 10.1186/s40364-023-00541-1

**Published:** 2023-11-09

**Authors:** Jie Mei, Leiyu Hao, Xiaorui Liu, Guangshun Sun, Rui Xu, Huiyu Wang, Chaoying Liu

**Affiliations:** 1 Department of Oncology, Wuxi People’s Hospital Affiliated to Nanjing Medical University, Wuxi, 214023 China; 2 Department of Physiology, Nanjing Medical University, Nanjing, 211166 China; 3 School of Pediatrics, Nanjing Medical University, Nanjing, 211166 China; 4 Department of General Surgery, Wuxi People’s Hospital Affiliated to, Nanjing Medical University, Wuxi, 214023 China

**Correction: Biomark Res 7, 16 (2019)**

**https://doi.org/10.1186/s40364-019-0168-9**

The original article [1] contains the following errors:

1. The ordinate scale is displayed incorrectly in Fig. 2D-F.
2. Fig. 3E was duplicated over Fig. 3F.
3. Table 3 contained data in the ‘HR’, ‘95% CI’, and ‘*P*-value’ columns of the ‘Adjuvant chemotherapy’ and ‘Non-chemotherapy’ rows that should have been deleted.

**Fig. 2:**
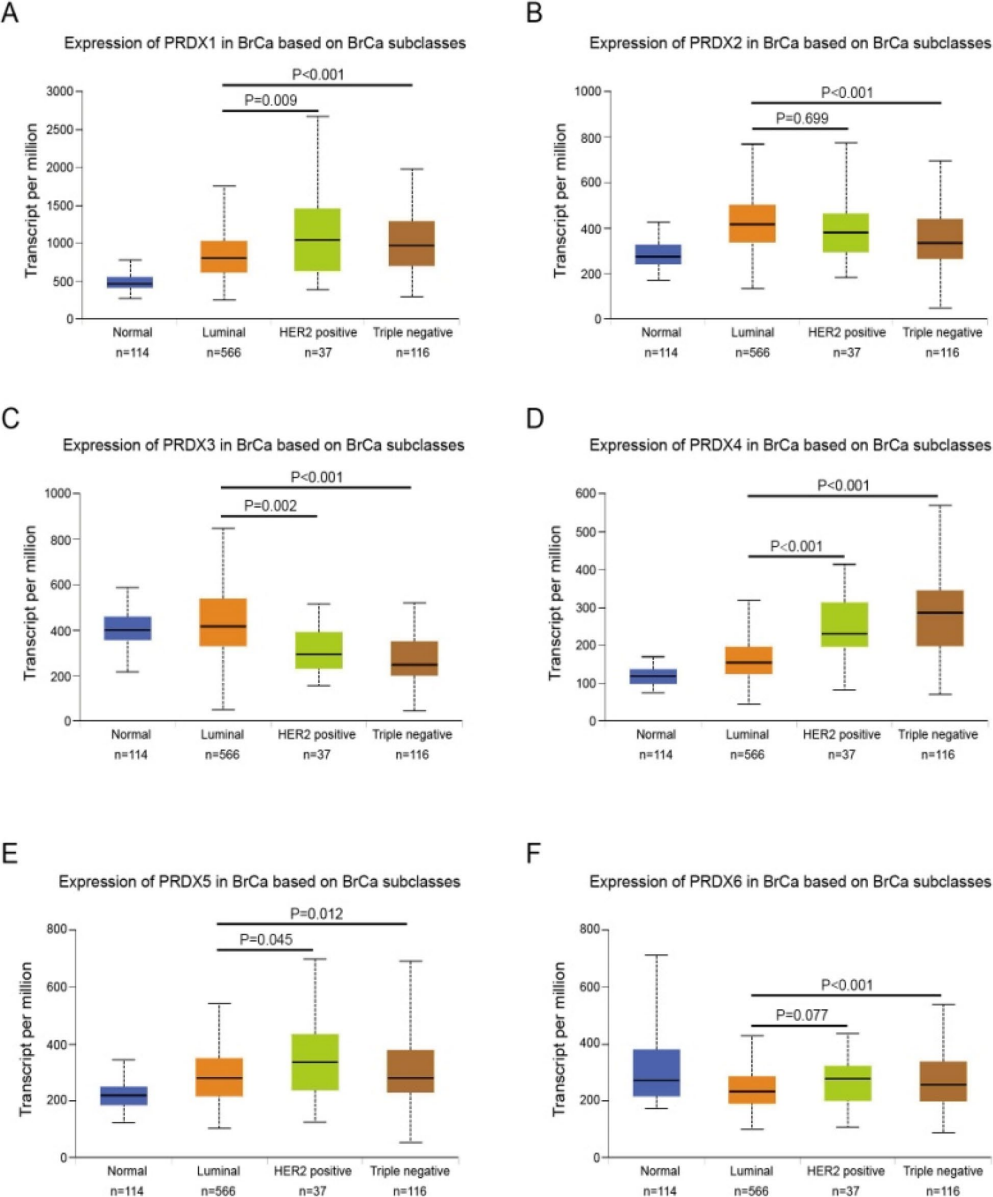
Transcriptional levels of PRDXs in various BrCa subclasses. The transcriptional level of PRDXs in BrCa patients with different subclasses, PRDXs mRNA was significantly downregulated (PRDX3) or upregulated (other PRDXs) in HER2-positive and triple-negative BrCa tissues compared with luminal BrCa tissues. **a** PRDX1. **b** PRDX2. **c** PRDX3. **d** PRDX4. **e** PRDX5. **f** PRDX6

**Fig. 3:**
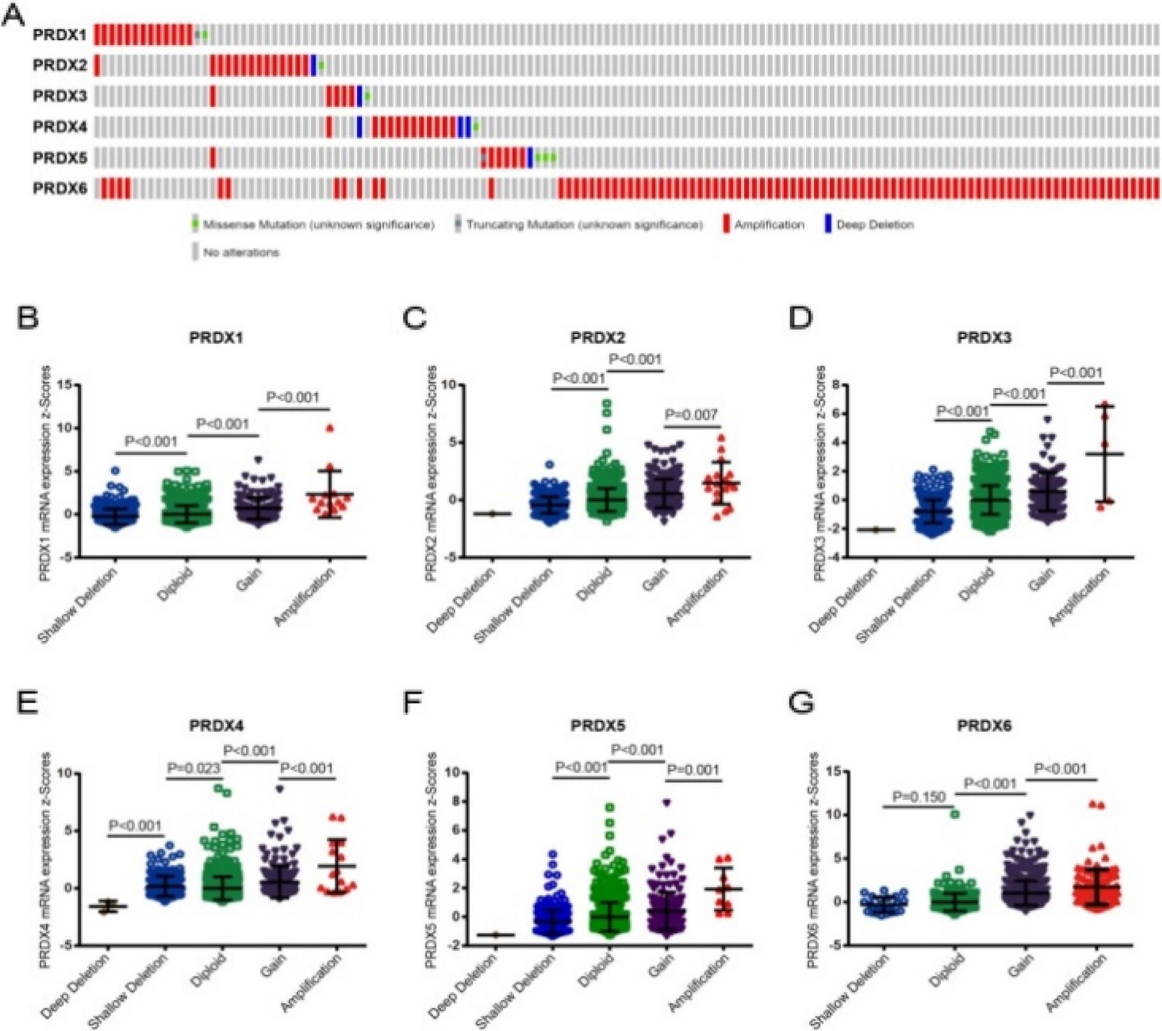
Correlation between the genetic alterations of PRDXs and mRNA levels in BrCa tissues. **a** Oncoprint in cBioPortal database exhibited the proportion and distribution of specimens with genetic alterations in PRDXs. The Figure was cropped on the right to exclude specimens without any alterations. **b**-**g** Copy gain (gain and amplification) of PRDXs was associated with notably upregulated PRDXs mRNA levels compared with the copy-neutral (diploid) and copy-loss (shallow deletion and deep deletion) cases. **b** PRDX1. **c** PRDX2. **d** PRDX3. **e** PRDX4. **f** PRDX5. **g** PRDX6

**Table 3:** Association between prognostic value of PRDXs mRNA expression and various chemotherapies in BrCa

Chemotherapies	Cases	HR	95%CI	*P* value	Cases	HR	95%CI	*P* value
		OS			RFS			
**PRDX1**								
Adjuvant chemotherapy	163	1.85	1.01–3.40	0.044	594	1.05	0.78–1.42	0.734
Neoadjuvant chemotherapy	156	0.74	0.34–1.60	0.448	223	1.19	0.69–2.03	0.539
Non-chemotherapy	549	1.13	0.79–1.60	0.502	1873	1.22	1.03–1.44	0.019
**PRDX2**								
Adjuvant chemotherapy	163	2.02	1.09–3.73	0.023	594	1.33	0.98–1.80	0.064
Neoadjuvant chemotherapy	156	1.41	0.66–3.02	0.375	223	1.24	0.72–2.16	0.438
Non-chemotherapy	549	1.14	0.80–1.62	0.483	1873	1.07	0.90–1.26	0.444
**PRDX3**								
Adjuvant chemotherapy	163	2.03	1.10–3.73	0.021	594	0.80	0.59–1.08	0.148
Neoadjuvant chemotherapy	156	1.05	0.49–2.23	0.906	223	0.95	0.55–1.65	0.851
Non-chemotherapy	549	0.79	0.56–1.12	0.185	1873	0.84	0.71–1.00	0.043
**PRDX4**								
Adjuvant chemotherapy	163	1.97	1.07–3.65	0.027	594	1.02	0.75–1.38	0.906
Neoadjuvant chemotherapy	156	0.68	0.31–1.48	0.325	223	1.19	0.69–2.07	0.529
Non-chemotherapy	549	1.28	0.90–1.82	0.177	1873	1.37	1.16–1.62	0.000
**PRDX5**								
Adjuvant chemotherapy	0	/	/	/	255	0.50	0.30–0.81	0.005
Neoadjuvant chemotherapy	107	0.67	0.24–1.89	0.446	111	1.22	0.58–2.57	0.595
Non-chemotherapy	0	/	/	/	243	0.66	0.38–1.14	0.131
**PRDX6**								
Adjuvant chemotherapy	163	0.86	0.48–1.56	0.625	594	1.10	0.81–1.49	0.532
Neoadjuvant chemotherapy	156	0.72	0.33–1.55	0.395	223	1.09	0.63–1.88	0.769
Non-chemotherapy	549	1.33	0.94–1.88	0.110	1873	1.19	1.00–1.40	0.044

The corrected Fig. 2, Fig. 3, and Table 3 all appear ahead. The authors apologize for this error and state that this does not change the scientific conclusion in any way.
